# Mr. Clark, on the Stone

**Published:** 1806-12

**Authors:** 


### To the Editors of the Medical and Phyſical Journal.

GENTLEMEN,

SOME days since, I was consulted by a woman respecting an incontinence of urine, under which she had laboured for twelve months. The history she gave of herself was, that at the period above mentioned, after having experienced for some time, a difficulty in making water, she voided a large stone, which she produced. At first, I suspected that the pressure of the stone upon the posterior part of the meatus urinarius had produced inflammation and ulceration there, and that the urine was flowing into the vagina, out of that opening through which the stone had twelve-months before escaped; an occurrence sometimes met with after hard labours, during which a considerable and long continued pressure has been made upon the meatus urinarius. Perhaps there is no state of disease, in which the patient's safety is not concerned, more distressing than the one I am now describing; for the constant dribbling of the urine over the skin of the labia and thighs, excoriates and inflames it, and the smell renders the unhappy sufferer disgusting to all who come near her. As far as I have had an opportunity of observing, this complaint occurs more frequently after the operation for the stone in women and girls than in men, which may probably be owing to the want of support to the meatus urinarius in the former.

There are two very different cases of incontinence of urine. In the one, the urine passes through a fistulous opening, between the bladder and vagina; the other depends upon a want of tone in the passage, leading from the bladder, which want of tone sometimes depends upon too great previous dilatation of the canal.—For the first case, although the use of the flexible catheter has now and then been of service generally, little more can be done than wearing a sponge to absorb the moisture. The latter case may be sometimes cured. It being then of importance to ascertain the precise nature of the disease, I made an examination; which I had scarcely began, when I was almost satisfied there was no fistulous opening in the side of the urethra; for I could introduce with great ease the extremity of my finger into the natural orifice, which cannot be done in a state of health, probably therefore, never, when there is any opening higher up in the passage, communicating cating with the vagina, for no water passing in this case through it, the duct contracts. To satisfy myself completely, I introduced a female catheter into the bladder, but could not detect any part of it by my finger, when placed in the vagina. A small quantity of urine running off, when the extremity of the catheter had entered the bladder, strengthened my opinion. In this case, I wished much to make use of blisters to the sacrum, a practice which has sometimes succeeded. I did not, however, think it prudent, to adopt any measures which could increase irritation, until I had allayed that arising from the excoriated thighs and labia. With this view, I directed an astringent lotion, and when I see my patient again, I shall by the adoption of some active means, endeavour to get rid of this unpleasant complaint.

It has been frequently remarked, that a stone in the bladder of women, seldom arrives at such a size, as to require operation for its removal, because of the straightness, shortness, and capacity of the meatus urinarius, all of which circumstances are very favourable to the passage of a stone through it.

A Case is related by Tulpius, of an old woman, who voided a stone from her bladder, which weighed three ounces; and in the fourth volume of the Medical Essays and Observations, published by a Society in Edinburgh in 1737, a case is related of a stone weighing above half an ounce, passing through the meatus urinarius. There is a singular circumstance connected with the formation of this stone, which is, that its nucleus was a needle.

It would lead me into too wide a field to make any observations upon the nature or singularity of the substances which are found to form the nuclei of urinary calculi; it may be sufficient to say, that pieces of bougies, a hazelnut, &c. have been frequently found within the firmest urinary calculus. Nor do I here mean to enter into the analysis of urinary calculi. This has been given in a most accurate manner by many much better chemists than myself, to whose works and papers in the Transactions of learned Societies a reference may be made. The stone which came from my patient resembles, in size and shape, that mentioned and delineated in the volume of the Medical Essays above alluded to. In the latter case, as well as in that related by Tulpius, incontinence of urine was the consequence, but no mention is made of any attempt to relieve it.

From the appearance of the stone which came away from from my patient, I think there is good reason to believe, that it did not arrive at its present size in the bladder, but that its apical part descended into the meatus urinarius; that not finding its way readily through, it became a nucleus for additional stony matter, which continued to accumulate, until the disposition for dilatation in the canal was greater than the additional bulk made to the stone was quick; and that then it was expelled. The length of the stone is an inch and a half, and it measures in circumference more than two inches. The shape of the stone resembles a small penis, divested of its preputium. The apical part is smooth and compact in texture, but the basis is very irregular, and consists of several little granulated proportions, which are so soft and loosely connected with each other, that every trifling agitation in carrying, has rubbed them off. The calculus is very light for its size.—Incontinence of urine, the complaint so frequently left in these cases, calls for much attention from the surgeon, for as it springs from a variety of sources, the mode of relief must of course be different.

By observing the effects which certain substances produce, when applied to the surface of the body, or taken internally, we are enabled to apply them to the cure of disease. Strangury, the very converse of the above disease, is frequently produced by the external application, or internal exhibition of the cantharis. The knowledge of this fact, enables us sometimes to cure an incontinence of urine, either by the application of the fly in the form of plaister, or tincture to the sacrum, or by the exhibition of it internally. Perhaps the tincture is the most safe way of giving it, and the vehicle may be decoction of bark. It may be given in the dose of ten or twenty drops, and increased.

When incontinence of urine arises merely from want of tone in the neck of the bladder and meatus urinarius, and all applications are found useless, the patient may reap much benefit by the application of a compress upon the urinary passage. This prevents the escape of the urine, and can be removed, when the quantity becomes so considerable as to be inconvenient.—This compress has been called by surgical writers, the Jugum or Yoke, which in men, is placed upon the penis. In women, the same effect may be produced by a pessary worn in the vagina. In the latter, however, there is an objection, which does not obtain in men, that no pressure can well be made upon the meatus urinarius of a woman, without some being also made made upon the rectum behind, which would tend to increase that disposition to costiveness, which is too prevalent in the sex. But supposing that it answers the purpose in men, of preventing the escape of the urine by the penis, it is not therefore always to be resorted to.—It is a fact very well known, that newly formed parts give way more readily than originally formed parts. It may happen then when the parts have been healed, after the operation for the stone, that the pressure of the urine may make the cicatrix in the bladder and perinæum give way, and thus a fistulous opening in the perinæum may be produced, infinitely more difficult of cure than the recent wound after the operation. In this case then it is not admissible; an apparatus, adapted to receive the urine, is here the only resource; and if by any escaping, the surrounding parts should be excoriated, nothing will be found more effectual in relieving it than a cold infusion of green tea; and if there should be much irritation, a liniment, composed of equal parts of ung. sperm. ceta and ol. lini, with a few drops of aq. litharg. acetat.

